# Some Pneumococcal Serotypes Are More Frequently Associated with Relapses of Acute Exacerbations in COPD Patients

**DOI:** 10.1371/journal.pone.0059027

**Published:** 2013-03-11

**Authors:** Arnau Domenech, Carmen Ardanuy, Roman Pallares, Immaculada Grau, Salud Santos, Adela G. De la Campa, Josefina Liñares

**Affiliations:** 1 Department of Microbiology, Hospital Universitari de Bellvitge-IDIBELL-Barcelona University, Barcelona, Spain; 2 CIBERES (Ciber de Enfermedades Respiratorias). ISCIII, Madrid, Spain; 3 Department of Infectious Diseases, Hospital Universitari de Bellvitge-IDIBELL-Barcelona University, Barcelona, Spain; 4 Department of Pneumology, Hospital Universitari de Bellvitge-IDIBELL, Barcelona, Spain; 5 Centro Nacional de Microbiología, Instituto de Salud Carlos III, Majadahonda, Madrid, Spain; 6 Consejo Superior de Investigaciones Científicas, CSIC, Madrid, Spain; School of Medicine, Univ. Complutense, Spain

## Abstract

**Objectives:**

To analyze the role of the capsular type in pneumococci causing relapse and reinfection episodes of acute exacerbation in COPD patients.

**Methods:**

A total of 79 patients with 116 recurrent episodes of acute exacerbations caused by *S. pneumoniae* were included into this study (1995–2010). A relapse episode was considered when two consecutive episodes were caused by the same strain (identical serotype and genotype); otherwise it was considered reinfection.

Antimicrobial susceptibility testing (microdilution), serotyping (PCR, Quellung) and molecular typing (PFGE/MLST) were performed.

**Results:**

Among 116 recurrent episodes, 81 (69.8%) were reinfections, caused by the acquisition of a new pneumococcus, and 35 (30.2%) were relapses, caused by a pre-existing strain. Four serotypes (9V, 19F, 15A and 11A) caused the majority (60.0%) of relapses. When serotypes causing relapses and reinfection were compared, only two serotypes were associated with relapses: 9V (OR 8.0; 95% CI, 1.34–85.59) and 19F (OR 16.1; 95% CI, 1.84–767.20). Pneumococci isolated from relapses were more resistant to antimicrobials than those isolated from the reinfection episodes: penicillin (74.3% *vs*. 34.6%, p<0.001), ciprofloxacin (25.7% *vs.* 9.9%, p<0.027), levofloxacin (22.9% *vs.* 7.4%, p = 0.029), and co-trimoxazole (54.3% *vs.* 25.9%, p<0.001).

**Conclusions:**

Although the acquisition of a new *S. pneumoniae* strain was the most frequent cause of recurrences, a third of the recurrent episodes were caused by a pre-existing strain. These relapse episodes were mainly caused by serotypes 9V and 19F, suggesting an important role for capsular type.

## Introduction

Chronic obstructive pulmonary disease (COPD) is a major cause of morbidity and mortality in developed countries [1]. Approximately 50% of acute exacerbation episodes of COPD are caused by bacterial pathogens, mainly *Streptococcus pneumoniae*, *Haemophilus influenzae* and *Moraxella catarrhalis* [2]. The development of an acute exacerbation episode caused by *S. pneumoniae* is thought to be associated with the acquisition of a new strain, although scarce information is available [3].

Capsular type, the principal pneumococcal virulence factor, had been related to the ability of pneumococci to cause invasive disease or colonization [4]. However, the aetiological role of pneumococcal serotypes in relapse episodes of COPD patients remains to be determined.

The aims of this study were to analyse the relationship between serotype and genotype and the ability to cause relapse or reinfection episodes in patients with COPD. In addition, we have explored the influence of previous antimicrobial therapy in this occurrence.

## Results

### Epidemiological and clinical data

A total of 79 COPD patients were included in the study. Their mean age was 69 (SD ±6) years, and 77 (97.5%) of them were males. In terms of COPD severity there was 1 patient with mild (1.3%), 14 patients with moderate (17.7%), 18 patients with severe (22.8%) and 39 patients with very severe (49.4%) airflow obstruction. The GOLD status of the remaining 7 patients was not available.

Fifty-two patients had ≥1 reinfections (caused by pneumococci with different serotype and genotype), 16 had ≥1 relapses (caused by the same pneumococcus), and the remaining 11 patients had both relapses and reinfections. No differences were observed as regards the age of patients with relapse or reinfection (69.9±9 *vs.* 70.5±9, respectively), nor in terms of COPD severity (GOLD II: 71.7±13 *vs.* 69.2±8, respectively, GOLD III: 66.4±7 *vs.* 70.6±11, respectively; and GOLD IV: 69.1±9 *vs.* 69.4±8, respectively).

Finally, a total of 116 recurrent episodes from the 79 patients were studied; of these, 35 (30.2%) were relapses and 81 (69.8%) reinfections. The mean time between episodes was 166±96 days, being shorter among relapses (133±89 days) than among reinfections (181±96 days; *P* = 0.020). Table 1 shows the distribution of the episodes based on the mean time between episodes. When the consecutive episodes occurred during a period ≤120 days, significantly higher frequency of relapse episodes was observed (57.1% *vs*. 35.8%, *P* = 0.033). Whereas, when the period of time between episodes was higher than 240 days the frequency of reinfection episodes was higher (14.3% *vs.* 30.9%, *P* = 0.061).

**Table 1:** Distribution of the number of relapses and reinfections based on the time between episodes.

Time between episodes (days)	Relapses (n = 35)	Reinfections (n = 81)	P-value
≤120	20 (57.1%)	29 (35.8%)	**0.033**
121–240	10 (28.6%)	27 (33.3%)	0.613
>240	5 (14.3%)	25 (30.9%)	0.061

In 13 of the 35 (37.1%) relapses and in 31 of the 81 (38.3%) reinfections, pneumococcal strains were isolated together with other potential pathogens.

However, *P. aeruginosa* was more frequently isolated from relapses than from reinfections (17.1% *vs.* 7.4%, respectively; *P = *0.108), whereas *H. influenzae* was more closely associated with reinfections (2.9% *vs.* 23.5%; *P = *0.006).

### Serotyping and genotyping

Four of the 31 serotypes detected caused 60.0% of relapses. These serotypes were 9V (17.1%), 19F (17.1%), 15A (14.3%) and 11A (11.4%). The most frequent serotypes isolated from reinfections were 15A (8.6%), 16F (7.4%), 3 (6.2%) and 33F (6.2%). Statistically significant differences were only observed in two serotypes associated with relapses when compared with reinfections: 9V (OR 8.0; 95% CI, 1.34–85.59) and 19F (OR 16.1; 95% CI, 1.84–767.20) [Table 2].

**Table 2:** Serotypes causing relapse and reinfection episodes of acute exacerbations in COPD patients.

serotype	relapses (n = 35)	reinfections (n = 81)	OR	95%CI
9V	6 (17,1%)	2 (2.5%)	**8.00**	1.34–85.59
19F	6 (17,1%)	1 (1.2%)	**16.11**	1.84–767.20
15A	5 (14,3)%	7 (8.6%)	1.75	0.41–7.01
11A	4 (11.4%)	3 (3.7%)	3.32	0.53–23.95
3	3 (8.6%)	5 (6.2%)	1.42	0.21–7.82
6C	2 (5.7%)	0	infinite	0.44–infinite
22F	2 (5.7%)	3 (3.7%)	1.57	0.13–14.36
19A	2 (5.7%)	4 (4.9%)	1.17	0.10–8.59
35B	1 (2.9%)	1 (1.2%)	2.33	0.03–186.68
33F	1 (2.9%)	5 (6.2%)	0.45	0.01–4.24
23F	1 (2.9%)	2 (2.5%)	1.16	0.02–22.99
23A	1 (2.9%)	2 (2.5%)	1.16	0.02–22.99
14	1 (2.9%)	4 (4.9%)	0.57	0.01–6.03
16F	0	6 (7.4%)	0	0.00–1.93
7F	0	4 (4.9%)	0	0.00–3.51
38	0	4 (4.9%)	0	0.00–3.51
31	0	4 (4.9%)	0	0.00–3.51
Non-typeable	0	4 (4.9%)	0	0.00–3.51
6B	0	3 (3.7%)	0	0.00–5.63
10A	0	3 (3.7%)	0	0.00–5.63
35F	0	2 (2.5%)	0	0.00–12.39
23B	0	2 (2.5%)	0	0.00–12.39
Other serotypes	0	10^a^		

a Serotypes 1, 5, 9N/L, 12F, 15B/C, 17F, 18C, 24F, 29 and 34 were detected in only one reinfection episode.

Serotypes included in the polysaccharide pneumococcal 23-valent (23vPPV) accounted for 74.3% (n = 26) of relapses, and 56.8% (n = 46) of reinfections. Whereas, the coverage of the 10-valent (PCV-10) and 13-valent (PCV-13) pneumococcal conjugate vaccines in all relapses were 40.0% (n = 16) and 54.3% (n = 19), respectively; and the coverage of reinfections were 23.5% (n = 19) and 34.6% (n = 28), respectively.

Seventeen PFGE patterns (related with 14 sequence types) were observed among the relapses, with the most frequent clonal complexes (CC) being CC156^9V^ (22.9%), CC63^15A,19F^ (17.1%), CC88^19F^ (11.4%), CC81^19A,19F^ (8.6%) and CC260^3^ (5.7%). Among reinfections, 56 different PFGE patterns were observed, and the most frequent clones were CC63^15A^ (7.4%), CC30^16F^ (7.4%), CC717^33F^ (4.9%), CC156^9V^ (4.9%), CC42^23A,23F^ (3.7%), CC62^11A^ (3.7%), CC97^10A^ (3.7%), CC191^7F^ (3.7%) and CC260^3^ (3.7%).

Only the CC156^9V^ genotype was associated with relapses (OR 5.8; 95% CI, 1.61–20.73). Serotype 19F was genetically heterogeneous [CC88^19F^ (6.2%), CC81^19F^ (2.9%), and CC63^19F^ (2.9%)].

### Antimicrobial consumption and susceptibility

Table 3 shows the activity of nine antimicrobials against pneumococci isolated from relapses and reinfections. Resistance to betalactams, fluoroquinolones and co-trimoxazole was higher among the strains that caused relapses than among those causing reinfections (P<0.01).

**Table 3:** *In vitro* activity of nine antimicrobials against pneumococci isolated from relapses and reinfections

	Relapses (n = 35)						Reinfections (n = 81)						P-value^f^
Antibiotic	MIC_50_ (mg/L)	MIC_90_ (mg/L)	MIC range (mg/L)	% S	% I	% R	MIC_50_ (mg/L)	MIC_90_ (mg/L)	MIC range (mg/L)	% S	% I	% R	
Penicillin	0.5	4	≤0.03–4	25.7^a^	45.7	28.6	0.06	0.5	≤0.03–2	65.4^a^	28.4	6.2	0.000
				82.9^b^	17.1	0				98.8^b^	1.2	0	0.003
Cefotaxime	0.12	1	≤0.03–2	68.6^c^	28.6	2.9	0.05	0.5	≤0.03–1	92.6^c^	7.4	0	0.001
				97.1^d^	2.9	0				100^d^	0	0	0.302
Ciprofloxacin^e^	1	>32	≤0.5–>32	74.3	–	25.7	≤0.5	1	≤0.5–>32	90.1	–	9.9	0.027
Levofloxacin	≤0.5	>32	≤0.5–>32	77.1	2.9	20.0	≤0.5	1	≤0.5–>32	92.6	0	7.4	0.029
Tetracycline	≤2	>32	≤2–>32	54.3	2.9	42.8	≤2	>32	≤2–>32	70.4	0	29.6	0.205
Erythromycin	≤0.25	>32	≤0.25–>32	57.1	0	42.9	≤0.25	>32	≤0.25–>32	67.9	0	32.1	0.266
Clindamycin	≤0.25	>32	≤0.25–>32	57.1	0	42.9	≤0.25	>32	≤0.25–>32	65.4	0	34.6	0.396
Chloramphenicol	≤2	>8	≤2–>8	88.6	–	11.4	≤2	8	≤2–>8	93.8	–	6.2	0.081
Co-trimoxazole	2/38	>4/76	≤0.5/9.5–>4/76	45.7	5.7	48.6	≤0.5/9.5	>4/76	≤0.5/9.5–>4/76	74.1	1.2	24.7	0.000

Clinical Laboratory Standard Institute (CLSI) breakpoints: ^a^Penicillin oral breakpoints: susceptible ≤0.06 mg/L, intermediate 0.12–1 mg/L and resistant ≥2 mg/L. ^b^Penicillin parenteral (non-meningitis) breakpoints: susceptible ≤2 mg/L, intermediate 4 mg/L and resistant ≥8 mg/L. ^c^Cefotaxime (meningitis) breakpoints: susceptible ≤0.5 mg/L, intermediate 1 mg/L and resistant ≥2 mg/L. ^d^Cefotaxime parenteral (non-meningitis) breakpoints: susceptible ≤1 mg/L, intermediate 2 mg/L and resistant ≥4 mg/L. ^e^Non-susceptibility to ciprofloxacin breakpoint MIC ≥4 mg/L and susceptibility breakpoint ≤2 mg/L. ^f^ *P*-value comparing susceptible strains.

The most frequent antimicrobials consumed by these patients during the episode prior to reinfection or relapse (n = 116) were beta-lactams (49.2%), fluoroquinolones (25.9%) or both (5.2%). Consumption of fluoroquinolones during the previous episode was higher in relapses than in reinfections (40.0% *vs.* 19.8%, respectively; *P* = 0.02), whereas, no differences in the betalactams consumption was observed (25.7% of relapses and 27.1% of reinfections; *P* = 0.872).

## Discussion

Capsular type is known to play an important role in the invasiveness of pneumococcal strains [4]. Thus, some serotypes have been associated with invasive pneumococcal disease or with acute exacerbations of COPD [5]. Although the isolation of a new pneumococcal strain has been associated with a significantly increased risk of a new acute exacerbation [2], little information is available about the persistence of *S. pneumoniae* isolates.

Our results agree with other reports in which reinfection through acquisition of a new strain was the most frequent cause of acute exacerbation episodes [3]. However, our study shows that a third of these recurrences were caused by a persistent pneumococcal strain, suggesting that in COPD patients the persistence of the same strain could be underestimated.

Although in 12 of 35 relapses *S. pneumoniae* was isolated together with another pathogen (*P. aeruginosa, H. influenzae*, *M. catarrhalis* or *Staphylococcus aureus*) the role of *S. pneumoniae* in causing the acute exacerbation episodes is supported by the high predominance of Gram positive diplococci in the Gram stain of a good quality sputum sample.

Our results show that serotypes 9V and 19F were associated with relapses, suggesting that serotype could play an important role in the persistence of pneumococcal isolates. In addition, differences in genotype distribution were also detected. All isolates that expressed the serotype 9V belonged to the Spain^9V^-CC156; hence, this clone was associated with the relapse episodes. In contrast, several genotypes expressed the serotype 19F and none of them was significantly associated with relapses. These results suggest that capsular type, rather than genetic background, may play an important role in the persistence of pneumococci among COPD patients.

Most of the patients included in the study had severe or very severe COPD, suffering frequent episodes of acute exacerbation and they received multiple antibiotic courses [6]. Although there were no differences in the betalactam consumption among groups, the betalactam resistance rates were higher among relapse episodes. This finding could be explained because relapse episodes were caused by few multi-resistant clones (mainly CC156 and CC88). Whereas, strains causing reinfection episodes showed a higher genetic diversity including penicillin-susceptible and -resistant clones. However, we found an association between fluorquinolone consumption and development of resistance. In fact, the development of fluorquinolone resistance during or after an antimicrobial course has been largely described in the literature [7]–[8].

The proportion of serotypes covered by the 23vPPV vaccine was high, especially those causing relapses. Unfortunately, vaccination data of patients included in the present study was not available; however, its protective efficacy in COPD populations is controversial since COPD adults respond differently than the general adult population, due to their impaired antibody response to the vaccine, the colonization of the lower respiratory tract, or the frequent use of inhaled corticosteroids [9]. In the other hand, conjugate vaccines (PV10 and PCV13) vaccine, which have an enhanced immunity potential, could prevent the 40% and a half of the overall relapse episodes, respectively.

The major limitations of our study are the low number of relapse episodes, and also that it is a retrospective study. Nonetheless, our study provides new data about the association of certain serotypes with the persistence of pneumococci and the ability to some clones, especially Spain^9V^-CC156, to cause relapse episodes. In addition, our study suggests that new episodes that occurred within the first 3 months after a previous one, had higher probability to be caused by the same pneumococcal strain and this fact could help to give an adequate empirical therapy.

Further studies with a high number of recurrent episodes are now needed to investigate not only the role of capsular type in relapses of acute exacerbations, but also whether the pneumococcal conjugate vaccine 13 could be beneficial for COPD patients.

## Methods

### Study design

Pneumococci and other potential pathogens isolated from sputum samples were prospectively collected into our laboratory between 1995 and 2010, and were frozen at −80°C for further analysis. Only pneumococci isolated from good quality sputum were considered (<10 squamous cells and >25 leucocytes per low-power field), with a predominance of Gram positive diplococci.

All COPD patients (n = 79) with two or more acute exacerbation episodes and seen at the Bellvitge University Hospital during the study period were included, after retrospective review of their computerized medical charts. Only those consecutive acute exacerbations which lasted for between four weeks and one year were included in the study.

The severity of airflow obstruction was categorized according to the Global Initiative for Chronic Obstructive Lung Disease (GOLD) criteria [10].

An acute exacerbation of COPD was defined as any sustained increase in respiratory symptomatology compared with the baseline situation requiring an increase in regular medication and hospital treatment. A ‘relapse’ episode was defined as two or more consecutive acute exacerbations caused by the same pneumococcus (identical serotype and genotype). When the consecutive episodes were caused by pneumococci with different serotype and Pulsed Field Gel Electrophoresis (PFGE) pattern they were defined as ‘reinfection’.

### Ethical statement

This study and publication of the results were approved by the ‘Comité Ètic d'Investigació Clínica del Hospital Universitari de Bellvitge’ and the written or oral informed consent was considered not necessary, because the source of bacterial isolates was anonymized and the study was retrospective.

### Serotyping and genotyping

Serotyping was performed by multiplex PCR, using a previously described methodology [11]. All isolates were genotyped by PFGE. Multi Locus Sequence Typing (MLST) was performed on all relapse isolates in order to confirm the identity of the isolates [12]–[13].

### Antimicrobial susceptibility

Antimicrobial susceptibility was tested by microdilution (STRHAE, Sensititre™), following the Clinical Laboratory Standards Institute (CLSI) criteria [14]. The ciprofloxacin MIC of resistant strains (MIC ≥4 mg/L) was confirmed by E-test. *S. pneumoniae* ATCC49619 was used as the control strain.

### Statistical analysis

Statistical analyses were carried out using SPSS 18 for Windows. The odds ratios (OR) and 95% confidence intervals (CI) were calculated, and Fisher's exact test was used when appropriate. Two-sided *P* values <0.05 were considered statistically significant.
